# Daratumumab with lenalidomide and dexamethasone in relapsed or refractory multiple myeloma patients – real world evidence analysis

**DOI:** 10.1007/s00277-023-05188-4

**Published:** 2023-04-24

**Authors:** Martin Stork, Ivan Spicka, Jakub Radocha, Jiri Minarik, Tomas Jelinek, Alexandra Jungova, Petr Pavlicek, Lenka Pospisilova, Frantisek Sedlak, Jan Straub, Tomas Pika, Zdenka Knechtova, Anna Fidrichova, Ivanna Boichuk, Sabina Sevcikova, Vladimir Maisnar, Roman Hajek, Ludek Pour

**Affiliations:** 1 Department of Internal Medicine, Hematology and Oncology, Faculty of Medicine, University Hospital Brno, Masaryk University, Brno, Czech Republic; 2 1st Medical Department – Clinical Department of Hematology of the First Faculty of Medicine, General Teaching Hospital Charles University, Prague, Czech Republic; 3 4th Department of Medicine – Hematology, Faculty of Medicine, Charles University Hospital, Hradec Kralove, Czech Republic; 4 Department of Hemato-Oncology, Faculty of Medicine and Dentistry, University Hospital Olomouc, Palacky University Olomouc, Olomouc, Czech Republic; 5 Department of Hematooncology, Faculty of Medicine, University Hospital Ostrava, University of Ostrava, Ostrava, Czech Republic; 6 Hematology and Oncology Department, Charles University Hospital, Pilsen, Czech Republic; 7 Department of Internal Medicine and Hematology, University Hospital Kralovske Vinohrady, Prague, Czech Republic; 8 Institute of Biostatistics and Analyses, Ltd., Brno, Czech Republic; 9 Babak Myeloma Group, Department of Pathophysiology, Faculty of Medicine, Masaryk University, Brno, Czech Republic

**Keywords:** Multiple myeloma, Treatment, Response rate, Relapse

## Abstract

We performed real world evidence (RWE) analysis of daratumumab, lenalidomide and dexamethasone (Dara-Rd) versus lenalidomide and dexamethasone (Rd) treatment in relapsed/refractory multiple myeloma patients (RRMM). In total, 240 RRMM patients were treated with Dara-Rd from 2016 to 2022 outside of clinical trials in all major Czech hematology centers. As a reference, 531 RRMM patients treated with Rd were evaluated. Patients’ data were recorded in the Czech Registry of Monoclonal Gammopathies (RMG). Partial response (PR) or better response (ORR) was achieved in significantly more patients in Dara-Rd than in Rd group (91.2% vs. 69.9%; p < 0.001). The median progression free survival (PFS) was 26.9 months in the Dara-Rd and 12.8 months in the Rd group (p < 0.001). Median overall survival (OS) was not reached in the Dara-Rd compared to 27.2 months in the Rd group (p = 0.023). In patients with 1–3 previous treatment lines, there was significant PFS benefit of Dara-Rd compared to Rd (median PFS not reached vs. 13.2 months; p < 0.001). In patients with > 3 previous treatment lines, there was no significant PFS benefit of Dara-Rd treatment (7.8 months vs. 9.9 months; p = 0.874), similarly in patients refractory to PI + IMIDs (11.5 months vs. 9.2 months; p = 0.376). In RWE conditions, the median PFS in RRMM patients treated with Dara-Rd is shorter when compared to clinical trials. In heavily pretreated RRMM patients, efficacy of Dara-Rd treatment is limited; best possible outcomes of Dara-Rd are achieved in minimally pretreated patients.

**Supplementary Information:**

The online version contains supplementary material available at 10.1007/s00277-023-05188-4.

## Introduction

In the last two decades, multiple myeloma (MM) treatment underwent significant progress [1]. The use of anti-CD38 monoclonal antibodies (mAbs) together with proteasome inhibitors (PI) and immunomodulatory drugs (IMIDs) became an emerging treatment modality with remarkable results. In clinical trials, anti-CD38 mAbs demonstrated high efficacy both in relapsed and newly diagnosed MM patients [2–6].

Daratumumab is the first widely used anti-CD38 mAb [7]. By binding to CD38 antigen on MM cells surface, daratumumab promotes apoptosis of MM cells and activates immune mechanisms (antibody-dependent cellular cytotoxicity (ADCC), antibody dependent cellular phagocytosis (ADCP) and complement dependent cytotoxicity (CDC)) leading to MM cell death [8, 9]. Immunomodulatory effect of daratumumab was also described to effect T-cells [10, 11]. In the first-in-human use, daratumumab monotherapy achieved response in 38% of heavily pretreated patients [12]. In preclinical tests, synergy of daratumumab and IMIDs was demonstrated [13]. Based on these results, in the phase III clinical trial POLLUX, daratumumab in combination with lenalidomide and dexamethasone (Dara-Rd) was compared to lenalidomide and dexamethasone (Rd). Dara-Rd regimen achieved deep responses (30.4% patients with negative minimal residual disease (MRD)) even in relapsed or refractory MM (RRMM) patients, and significantly prolonged median progression free survival (mPFS) to 45.5 months when compared to Rd alone (mPFS 17.5 months, p < 0.001) [2, 14]. Thus, Dara-Rd became a new standard-of-care for RRMM in many European countries [1, 15, 16].

However, randomized clinical trials (RCT) describe data different from real world evidence (RWE) conditions. In the RCT for MM, important subgroups of patients are often neglected. These subgroups are characterized with an aggressive disease course (extramedullary plasmacytoma, disease refractory to specific drugs, hyperviscosity with necessity of plasmapheresis, myeloma induced kidney failure, etc.) or significant comorbidities [17, 18].

With respect to these differences, we analyzed the outcomes of Dara-Rd regimen in real-world clinical conditions to define which patients benefit from Dara-Rd treatment the most.

### Patients and methods

Our study is a multicentric real-life retrospective study carried out in major hematologic centers in the Czech Republic between 2016 and 2022. Patients represent real world RRMM population treated with best available treatment at the time.

Data from patients treated in Dara-Rd and Rd groups were collected from the Registry of Monoclonal Gammopathies (RMG) of the Czech Myeloma Group. In total, 240 RRMM patients treated with Dara-Rd and 531 RRMM patients treated with Rd were enrolled. Only one patient from the Dara-Rd group was enrolled in 2016, the rest of the cohort was enrolled from 2019 to 2022. Patients in the Rd group were enrolled from 2016 to 2019, when Rd was the golden standard for RRMM patients’ treatment. Patients treated with Rd before 2016 were excluded for historical lenalidomide reimbursement rules in the Czech Republic (after cumulative dose of 4200 mg, lenalidomide treatment had to be stopped). Patients treated with Rd after 2019 were also censored, as modern lenalidomide-based triplets (with daratumumab, ixazomib or carfilzomib) were used in the Czech Republic as a new golden standard. Thus, after 2019, patients treated in the Czech Republic only with Rd were mostly palliative. Enrolling them could seriously bias our results, favoring Dara-Rd group. All enrolled patients were treated outside of clinical trials. All patients provided informed consent for participation in the study according to the declaration of Helsinki.

Patients in Dara-Rd group received standard dosing of Daratumumab 16 mg/kg intravenous or 1800 mg subcutaneous equivalent [19] on day 1, 8, 15, 22 in cycle 1–2, day 1, 15 in cycles 3–6, and day 1 at cycles 7 and more; lenalidomide 25 mg on days 1 through 21, and dexamethasone 20–40 mg on days 1, 8, 15 and 22 in 28-day cycles. Patients in Rd group received lenalidomide 25 mg on days 1 through 21 and dexamethasone 20–40 mg on days 1, 8, 15 and 22 in 28-day cycles. Reduction of lenalidomide or dexamethasone was allowed according to physicians’ decision. Patients in Dara-Rd arm had corticosteroid-based premedication according to institutional guidelines before daratumumab administration.

All patients were required to use thromboprophylaxis and herpes zoster prophylaxis per institutional guidelines. Cytogenetic aberrations were evaluated at the time of newly diagnosed multiple myeloma (NDMM).

### Assessments

All the data were recorded in the RMG. The endpoints were assessed based on the International Myeloma Working Group (IMWG) response criteria, incorporating an additional category of minimal response. Survival intervals progression free survival (PFS), duration of response (DOR) and overall survival (OS) were assessed from Dara Rd/Rd treatment beginning.

### Statistical analysis

Depending on the nature of the data, suitable methods for description and statistical testing were selected. Categorical variables were described using absolute and relative frequencies and continuous variables by median complemented with 5^th^ and 95^th^ percentile. In accordance with data continuity (categorical x continuous), Pearson Chi-Square (resp. Fisher's exact test in case of non-meeting criteria) or Mann–Whitney U test was used to examine the association between selected variables and treatment regimen. Event-free survival (PFS, DOR and OS) was assessed using the Kaplan–Meier methodology, and statistical significance of differences in survival between subgroups was assessed using the log-rank test. All statistical tests were performed at a significance level of α = 0.05 (all tests two-sided). The analysis was performed in SPSS software (IBM Corp. Released 2016. IBM SPSS Statistics for Windows, Version 25Armonk, NY: IBM Corp.) and software R version 3.4.2 (www.r-project.org).

## Results

### Patients and treatment

Altogether, 240 patients were treated with Dara-Rd regimen and 531 patients with Rd regimen. Median age was 66.0 years (5^th^–95^th^ percentile 45.9–77.7) in the Dara-Rd group and 71.1 years (5^th^–95^th^ percentile 54.1–82.6) in the Rd group (p < 0.001). There was a comparable number of patients with high-risk cytogenetic aberrations (HR-CA; t(4;14), t(14;16), del(17p)) in both groups (28.0% (37/132) vs. 29.4% (60/204), p = 0.806).

The median of previous treatment lines was comparable between both groups (1 [95% CI: 1–4] vs. 1 [95% CI: 1–4], p = 0.090). There were significantly more patients exposed to PI + IMIDs (64.2% (154/240) vs. 45.8% (243/531), p < 0.001) in the Dara-Rd group. Number of PI + IMIDs refractory patients were comparable in both groups (29.6% (71/240) vs. 32.2% (171/531), p = 0.503. The median follow-up from Dara-Rd vs. Rd treatment initiation was 13.5 months [95% CI: 1.3–26.6] in the Dara-Rd group and 23.7 months [95% CI: 1.5–59.7] in the Rd group. Baseline characteristics of patients are summarized in Table 1.

**Table 1:** Characteristics of Dara-Rd vs. Rd patients

	Rd		Dara-Rd		p-value
	N	%	N	%	
Sex	531	100%	240	100%	
Woman	267	50.3%	108	45.0%	0.186
Man	264	49.7%	132	55.0%	
Age at treatment initiation [years]	531	100%	240	100%	
< 65	127	23.9%	106	44.2%	< 0.001
65–75	263	49.5%	113	47.1%	
> 75	141	26.6%	21	8.8%	
median (5th–95th perc.)	71.1	54.1–82.6	66.0	45.9–77.7	< 0.001
ECOG PS at treatment initiation	506	100%	238	100%	
PS 0	85	16.8%	58	24.4%	< 0.001
PS 1	250	49.4%	143	60.1%	
PS 2	139	27.5%	34	14.3%	
PS 3–4	32	6.3%	3	1.3%	
ISS at treatment initiation	426	100%	200	100%	
Stage 1	154	36.2%	92	46.0%	0.037
Stage 2	150	35.2%	53	26.5%	
Stage 3	122	28.6%	55	27.5%	
Cytogenetic risk at diagnosis	204	100%	132	100%	
standard	144	70.6%	95	72.0%	0.806
high	60	29.4%	37	28.0%	
Number of previous lines of therapy	531	100%	240	100%	
1 previous line	341	64.2%	173	72.1%	0.045
2–3 previous lines	161	30.3%	52	21.7%	
> 3 previous lines	29	5.5%	15	6.3%	
median (5th–95th perc.)	1	1.0–4.0	1	1.0–4.0	0.090
Previous treatment by:	531	100%	240	100%	
Proteasome inhibitors (PI)	497	93.6%	234	97.5%	0.034
Immunomodulatory drugs (IMiD)	271	51.0%	159	66.3%	< 0.001
PI + IMiD	243	45.8%	154	64.2%	< 0.001
Transplantation	178	33.5%	161	67.1%	< 0.001
Refractory in previous treatment to:	531	100%	240	100%	
Proteasome inhibitors (PI)	155	29.2%	64	26.7%	0.491
Immunomodulatory drugs (IMiD)	69	13.0%	45	18.8%	0.038
Lenalidomide	11	2.1%	24	10.0%	< 0.001
PI + IMiD	171	32.2%	71	29.6%	0.503
Plasmacytoma	531	100%	240	100%	
no	406	76.5%	166	69.2%	0.022
Found in NDMM	66	12.4%	48	20.0%	
Developed in RRMM	59	11.1%	26	10.8%	
Length of therapy [months]	466	100%	100	100%	
median (5th–95th perc.)	7.4	1.2–33.9	7.5	0.7–23.7	0.254

### Response to treatment

According to IMWG criteria, treatment response was evaluable in 181 patients in the Dara-Rd group and 429 patients in the Rd group. Complete response (CR) or stringent CR (sCR) was achieved in 4.4% (8/181) of patients in Dara-Rd group, compared to 3.3% (14/429) of patients in Rd group. Very good partial response (VGPR) or better response was achieved in 66.8% (121/181) of patients in Dara-Rd group, compared to 27.5% (118/429) of patients in Rd group. Partial response (PR) or better response (ORR) was achieved in significantly more patients in Dara-Rd group than in Rd group (91.2% vs. 69.9%). Differences in treatment responses were statistically significant (p < 0.001), favoring Dara-Rd regimen (Table 2).

**Table 2:** Treatment results of Dara Rd vs. Rd

	Rd		Dara-Rd		p-value
	N	%	N	%	
Maximal response to treatment	429*	100,0%	181*	100,0%	
sCR, CR	14	3,3%	8	4,4%	< 0.001
VGPR	104	24,2%	113	62,4%	
PR	182	42,4%	44	24,3%	
MR	54	12,6%	9	5,0%	
SD	32	7,5%	6	3,3%	
PD	43	10,0%	1	0,6%	
Overal response rate (ORR)	429	100,0%	181	100,0%	
PR +	300	69,9%	165	91,2%	< 0.001
worse than PR	129	30,1%	16	8,8%	

^*^Only evaluable patients according to IMWG criteria

### Survival intervals

**Fig. 1:**
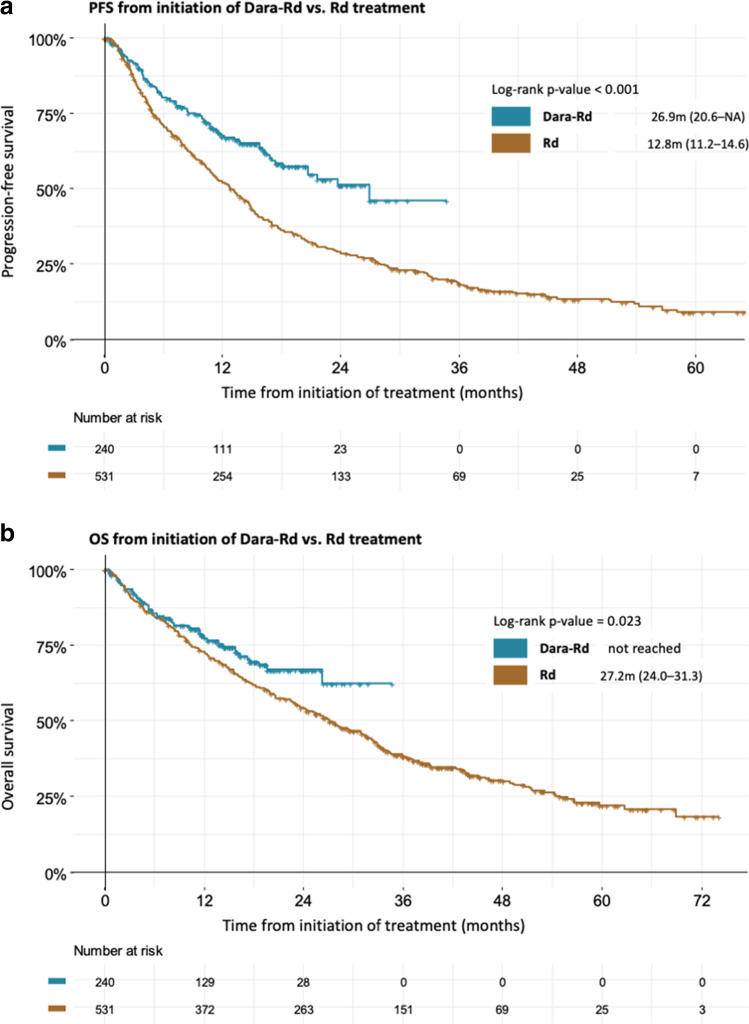
**A** Progression-free survival of Dara-Rd vs. Rd group. **B** Overall survival of Dara-Rd vs. Rd group

Median PFS was 26.9 months [95% CI: 20.6-NA] in the Dara-Rd group and 12.8 months [95% CI: 11.2–14.6] in the Rd group (HR: 1.81; [95% CI: 1.43–2.29]; p < 0.001) (Fig. 1A). Median OS was not reached in the Dara-Rd group compared to 27.2 months [95% CI: 24.0–31.3] in the Rd group (HR: 1.38; [95% CI: 1.05–1.83]; p = 0.023) (Fig. 1B).

### Progression free survival—subgroup analysis

**Fig. 2:**
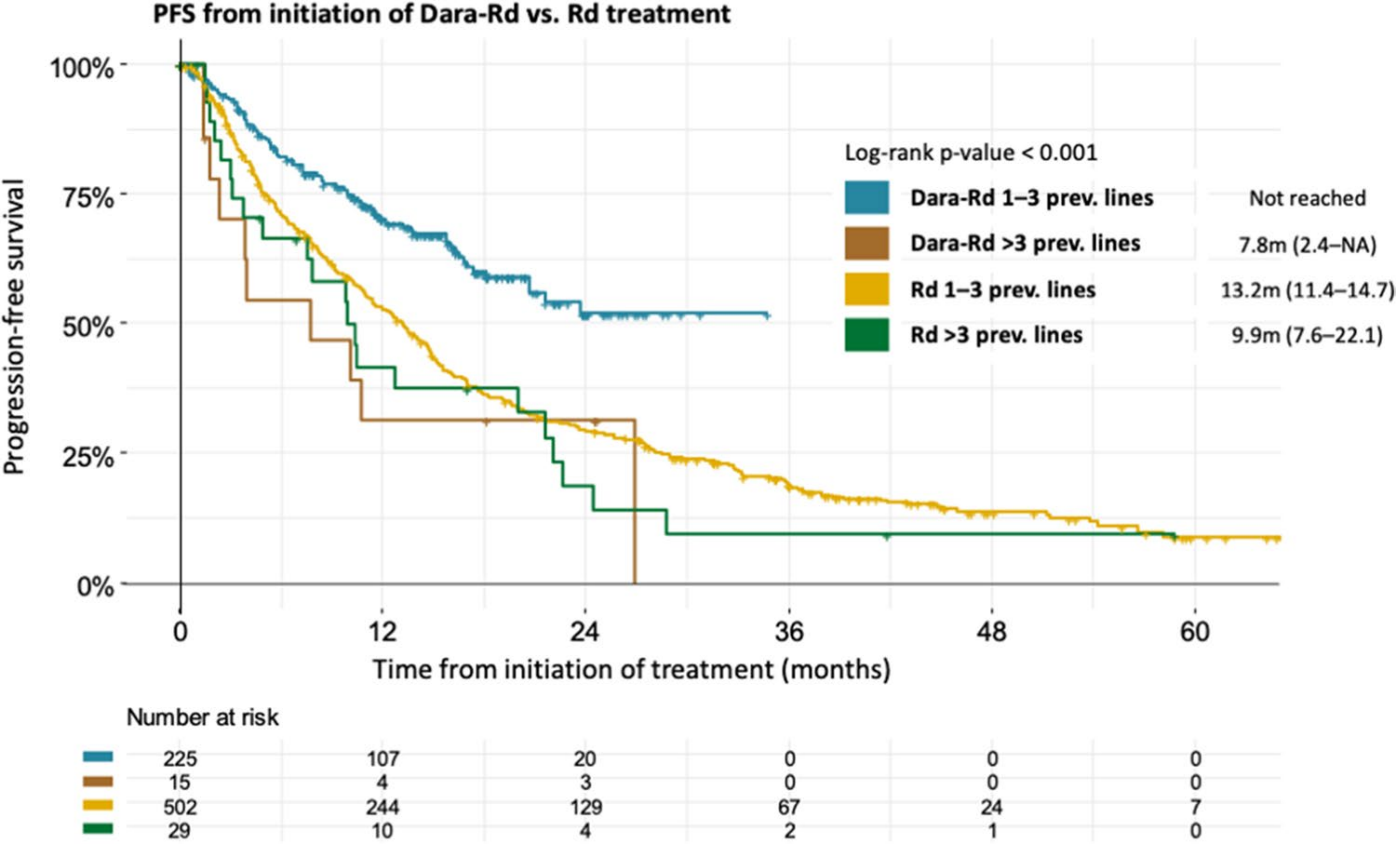
Progression-free survival of Dara Rd vs Rd patients with 1–3 previous treatment lines

In the subgroup of patients with 1–3 previous treatment lines, Dara-Rd treatment significantly prolonged PFS, when compared to Rd (Not reached vs. 13.2 months [95% CI: 11.4–14.7], HR: 1.94; [95% CI: 1.51–2.48]; p < 0.001). In patients with > 3 previous treatment lines, there was no significant PFS benefit of Dara-Rd treatment (7.8 months [95% CI: 2.4–NA] vs. 9.9 months [95% CI: 7.6–22.1], HR: 0.94; [95% CI: 0.44–2.00]; p = 0.874) (Fig. 2).

**Fig. 3:**
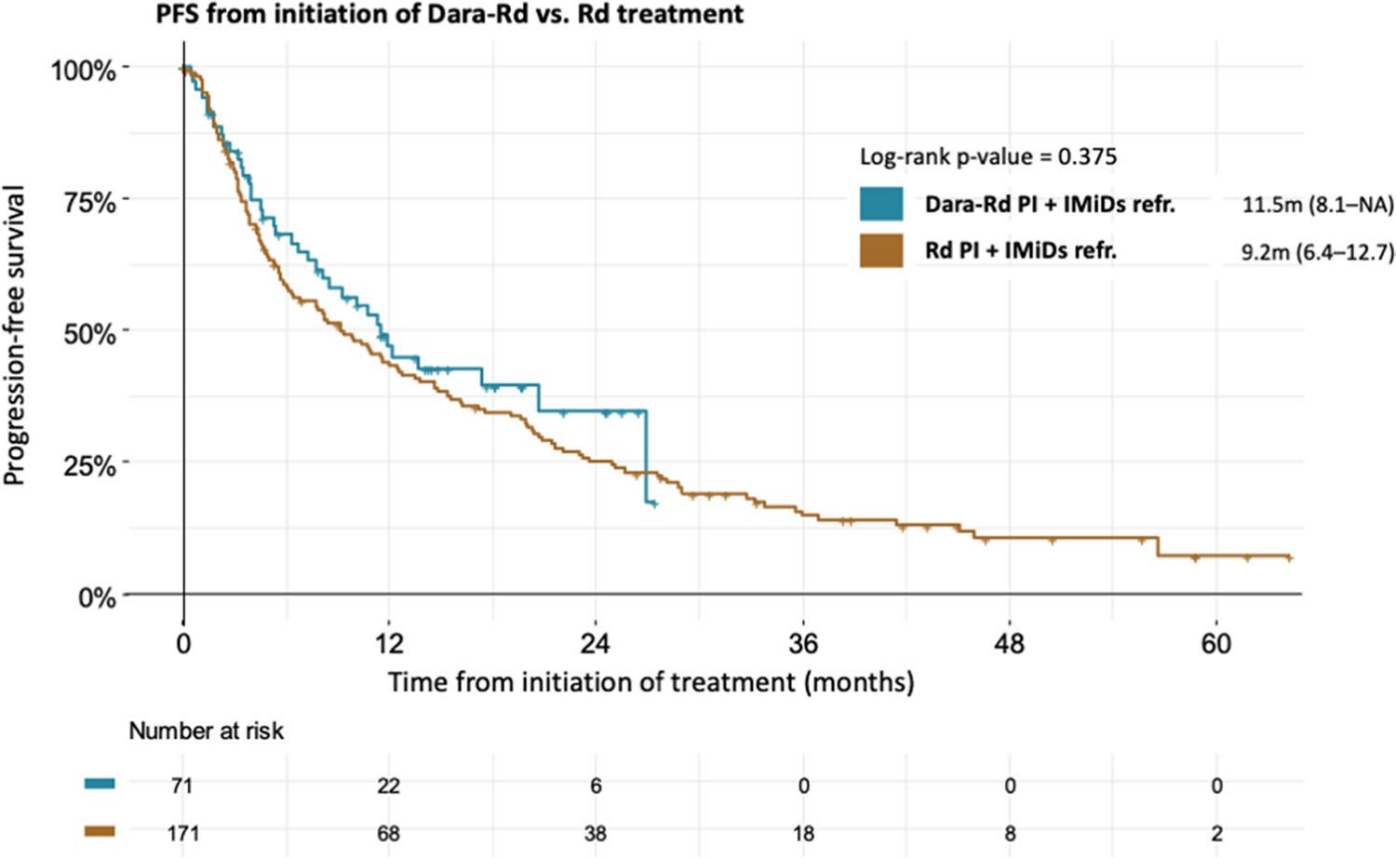
No significantly different PFS in PI/IMIDs refractory patients

In subgroup of patients who were refractory to PI + IMIDs, there was no significant PFS benefit of Dara-Rd treatment over Rd treatment (11.5 months [95% CI: 8.1–NA] vs. 9.2 months [95% CI: 6.4–12.7], HR: 1.18; [95% CI: 0.82–1.70]; p = 0.376) (Fig. 3).

In subgroup of patients who were refractory to lenalidomide, there was no significant PFS benefit of Dara-Rd treatment over Rd treatment (10.1 months [95% CI: 4.0–NA] vs. 12.7 months [95% CI: 4.9–NA], HR: 0.98; [95% CI: 0.39–2.45]; p = 0.961) (Supplementary Fig. 1).

In the subgroup of patients with HR-CA (t(4;14), t(14;16), del(17p)), there was no significant PFS benefit of Dara-Rd treatment (9.7 months [95% CI: 5.8–13.7] vs. 10.2 months [95% CI: 6.4–13.5], HR: 0.82; [95% CI: 0.51–1.33]; p = 0.428) (Supplementary Fig. 2), similarly to patients with gain(1q21) (13.8 months [95% CI: 9.9–20.7] vs. 10.2 months [95% CI: 6.9–12.8], HR: 1.33; [95% CI: 0.92–1.91); p = 0.129) (Supplementary Fig. 3). In the subgroup of RRMM patients with plasmacytoma found at the time of NDMM, Dara-Rd treatment prolonged PFS, when compared to Rd, but did not reach the level of statistical significance (23.6 months [95% CI: 10.8–NA] vs. 10.0 months [95% CI: 7.0–16.1], HR: 1.60; [95% CI: 0.95–2.69]; p = 0.080). In RRMM patients with plasmacytoma newly developed at disease relapse/progression, there was no significant PFS benefit of Dara-Rd treatment (9.9 months [95% CI: 3.9–16.5] vs. 6.4 months [95% CI: 4.4–12.8], HR: 0.85; [95% CI: 0.49–1.46]; p = 0.554) (Supplementary Fig. 4).

Dara-Rd treatment effect on PFS in different patients’ subgroups is summarized in Table 3.

**Table 3:** Association of Dara-Rd and Rd with survival in selected subgroups

Variable	Category	Rd	Dara-Rd	PFS – Univariable Cox model		
				HR*	95% CI	p-value
Total	Total	531	240	1.81	(1.43–2.29)	< 0.001
Age	≤ 75	390	219	1.75	(1.36–2.25)	< 0.001
	> 75	141	21	2.08	(0.97–4.49)	0.062
ISS	Stage 1	154	92	1.29	(0.85–1.96)	0.240
	Stage 2	150	53	2.23	(1.37–3.63)	0.001
	Stage 3	122	55	1.73	(1.11–2.71)	0.015
Creatinine level (µmol/l)	≤ 176	441	210	1.88	(1.45–2.43)	< 0.001
	> 176	77	30	1.44	(0.81–2.55)	0.219
ECOG	PS 0–1	335	201	1.76	(1.34–2.31)	< 0.001
	PS 2	139	34	1.35	(0.80–2.29)	0.267
	PS 3–4	32	3	0.52	(0.15–1.76)	0.293
Number of previous lines of therapy	1–3 previous lines	502	225	1.94	(1.51–2.48)	< 0.001
	> 3 previous lines	29	15	0.94	(0.44–2.00)	0.874
Plasmacytoma	no	406	166	2.31	(1.69–3.14)	< 0.001
	Found in NDMM	66	48	1.60	(0.95–2.69)	0.080
	Developed in RRMM	59	26	0.85	(0.49–1.46)	0.554
Cytogenetic risk at diagnosis	standard	144	95	1.88	(1.25–2.81)	0.002
	high	60	37	0.82	(0.51–1.33)	0.428
Gain(1q21)	no	158	89	1.96	(1.26–3.05)	0.003
	yes	111	84	1.33	(0.92–1.91)	0.129
Refractory to previous proteasome inhibitors (PI)	no	376	176	2.16	(1.60–2.91)	< 0.001
	yes	155	64	1.24	(0.84–1.82)	0.281
Refractory to immunomodulatory drugs (IMiD)	no	462	195	2.22	(1.68–2.93)	< 0.001
	yes	69	45	1.06	(0.67–1.70)	0.799
Refractory to lenalidomide	no	520	216	2.01	(1.56–2.58)	< 0.001
	yes	11	24	0.98	(0.39–2.45)	0.961
Refractory to PI + IMiD	no	360	169	2.28	(1.68–3.10)	< 0.001
	yes	171	71	1.18	(0.82–1.70)	0.376

^*^HR > 1 – Dara-Rd better; HR < 1 – Rd better

### Overall survival – subgroup analysis

In subgroup of patients who were refractory to PI + IMIDs, there was no significant OS benefit of Dara-Rd treatment over Rd treatment (19.6 months [95% CI: 13.7–NA] vs. 19.9 months [95% CI:14.6–24.4], HR: 1.07; [95% CI: 0.70–1.63]; p = 0.749). In the subgroup of patients with HR-CA, there was OS benefit of Dara-Rd treatment, but did not reach the level of statistical significance (13.7 months [95% CI: 10.1–NA] vs. 23.9 months [95% CI:15.3–29.6], HR: 0.72; [95% CI: 0.41–1.26]; p = 0.247). There was no significant OS benefit of Dara-Rd treatment over Rd in the subgroup of patients with gain(1q21) (not reached vs.24.6 months [95% CI: 17.8–31.4], HR: 1.10; [95% CI: 0.70–1.72); p = 0.690).

Dara-Rd treatment effect on OS in different patients’ subgroups is summarized in Table 4.

**Table 4:** Association of Dara-Rd and Rd with survival in selected subgroups

Variable	Category	Rd	Dara-Rd	OS – Univariable Cox model		
				HR*	95% CI	p-value
Total	Total	531	240	1.38	(1.05–1.83)	0.023
Age	≤ 75	390	219	1.28	(0.95–1.73)	0.104
	> 75	141	21	2.23	(0.81–6.12)	0.120
ISS	Stage 1	154	92	0.98	(0.58–1.66)	0.928
	Stage 2	150	53	1.53	(0.84–2.77)	0.164
	Stage 3	122	55	1.22	(0.74–1.99)	0.434
Creatinine level (µmol/l)	≤ 176	441	210	1.38	(1.01–1.89)	0.043
	> 176	77	30	1.38	(0.73–2.62)	0.321
ECOG	PS 0–1	335	201	1.20	(0.85–1.70)	0.289
	PS 2	139	34	1.09	(0.62–1.93)	0.769
	PS 3–4	32	3	0.56	(0.17–1.88)	0.349
Number of previous lines of therapy	1–3 previous lines	502	225	1.55	(1.14–2.09)	0.005
	> 3 previous lines	29	15	0.57	(0.26–1.25)	0.158
Plasmacytoma	no	406	166	1.72	(1.18–2.50)	0.004
	Found in NDMM	66	48	1.35	(0.73–2.47)	0.338
	Developed in RRMM	59	26	0.73	(0.39–1.39)	0.340
Cytogenetic risk at diagnosis	standard	144	95	1.56	(0.95–2.57)	0.081
	high	60	37	0.72	(0.41–1.26)	0.247
Gain(1q21)	no	158	89	1.34	(0.80–2.24)	0.272
	yes	111	84	1.10	(0.70–1.72)	0.690
Refractory to previous proteasome inhibitors (PI)	no	376	176	1.48	(1.03–2.12)	0.033
	yes	155	64	1.18	(0.76–1.85)	0.463
Refractory to immunomodulatory drugs (IMiD)	no	462	195	1.78	(1.26–2.50)	0.001
	yes	69	45	0.80	(0.47–1.36)	0.404
Refractory to lenalidomide	no	520	216	1.57	(1.16–2.14)	0.004
	yes	11	24	0.85	(0.31–2.32)	0.747
Refractory to PI + IMiD	no	360	169	1.60	(1.10–2.34)	0.015
	yes	171	71	1.07	(0.70–1.63)	0.749

^*^HR > 1 – Dara-Rd better; HR < 1 – Rd better

### Adverse events

Infusion related reactions (IRRs) gr. 2–3 after daratumumab administration were present in 13.3% (32/240) of patients. Higher grades of IRRs were not observed. Serious (gr. 3–4) adverse events (AEs) of Dara-Rd regimen were dominantly hematologic—neutropenia (50.9% (80/157)), anemia (14.9% (23/154)) and thrombopenia (16.3% (25/153)). Other serious (gr.3–4) non-hematologic AEs was infections 16.2% (29/179), thromboembolic disease (4.5% (7/154)), diarrhea (2.9% (5/171)), fatigue (1.8% (3/169)), polyneuropathy (1.3% (2/157), nausea (1.3% (2/156), decompensation of diabetes mellitus (1.0% (1/101)), rash (0.6% (1/158)) and anorexia (0.6% (1/157)). In the Dara-Rd group, there were 5 deaths related to infection complications. Mild (gr.1–2) AEs and comparison with Rd group is summarized in supplementary Table 1.

## Discussion

Novel drugs, such as daratumumab, isatuximab, carfilzomib and ixazomib, were carefully evaluated in RRMM patients in large multicentric RCT [2, 20]. Unluckily, population of MM patients eligible for RCT enrollment is significantly different from general MM patients’ population [17]. Therefore, there is a rapidly emerging importance of RWE analyses. Following a general trend of personalized medicine [21], in real-life conditions, it is necessary to differentiate which patients would benefit from specific treatment modality. Moreover, with various treatment options, proper timing of each treatment modality plays an important role [1]. Dealing with this issue, we performed a national RWE analysis of Dara-Rd treatment regimen.

Despite having an unselected patient population and not-evaluable treatment response in all patients, overall response to Dara-Rd treatment in our analysis was comparable to the POLLUX trial (91.2% vs. 92.9%) [14]. Low CR rate in our analysis is due to absence of routine BM evaluation in our RRMM patients, as the results would have had no practical impact on patients’ treatment course, and the treatment was until progression. On the other hand, survival intervals of Dara-Rd treatment were nearly half-time, when compared to the POLLUX trial. Main explanation is in the differences between the patients’ cohorts [14]. In our cohort, there was a higher proportion of patients with high-risk cytogenetic aberrations (28.0% vs. 15.0%), double refractory (PI + IMID) patients (29.6% vs 2.4%) and high proportion of patients with plasmacytomas (30.5%). In contrast with the POLLUX trial, we included 10% of lenalidomide refractory patients [14].

In our analysis, we found uncertain clinical benefit of Dara-Rd treatment in patients with more than 3 previous treatment lines. It is important to mention that our results may be influenced by low number of patients in this cohort. However, this finding was also shown in the POLLUX trial, when benefit from Dara-Rd was less pronounced in the more pretreated patients and vice versa (PFS: > 3 lines HR: 0.74 [CI 95% (0.24–2.26)] vs. HR: 0.42 [95% (0.30–0.58)]) [2]. Similarly, we found non-significant benefit of Dara-Rd treatment in patients refractory to PI, IMIDs or both. Our results are also unique for presence of lenalidomide refractory patients, while the POLLUX trial did not enroll them. These results show patients refractory to lenalidomide to have inferior outcome from the Dara-Rd treatment.

Overall, our analysis in accord with the POLLUX trial shows crucial role of Dara-Rd treatment timing, as the best effect is achieved in less pretreated patients [2]. These results are consistent with other RWE analysis, where the best results of daratumumab treatment were achieved in the first relapse (time to next treatment—25.9 months [22]). Similar results of Dara-Rd treatment in minimally pretreated patients were published by Italian [23, 24] or Spanish authors [25]. Results favoring the less pretreated patients were also shown in other triplet regimens, combining IMIDs and PI, like a pomalidomide-bortezomib-dexamethasone [26], or carfilzomib-lenalidomide-dexamethasone [27, 28]. These findings point to an actual unmet need for novel treatment strategies and molecular targets for multiple refractory MM patients instead of repeating of previously used drug classes [29–31].

In our analysis, we used HR-CA (del(17p), t(4;14) and t(14;16) based on the older classification from 2009 for better comparison with the POLLUX trial [32]. The benefit of Dara-Rd treatment in patients with HR-CA in our analysis was not significant, likewise in the patients with gain(1q21), nowadays recognized as a HR-CA [33]. This finding contrasts with the outcomes of the POLLUX trial where patients with HR-CA maintain PFS benefit by daratumumab treatment (HR: 0.43 [95% CI, 0.32–0. 57]) [2]. Similarly, there was PFS benefit in daratumumab with dexamethasone over bortezomib with dexamethasone (HR: 0.41 [95% CI, 0.21–0.83]) [34] and in combination of daratumumab—carfilzomib—dexamethasone over carfilzomib—dexamethasone alone (HR: 0.56 [95% CI, 0.34–0.93]) [35]. Interestingly, RCTs dealing with daratumumab in the front-line setting do not confirm clear benefit of daratumumab treatment in HR-CA patients [36–38]. This controversy only highlights necessity to consider HR-CA in a wider context of other high-risk factors, such as high LDH levels [39], extramedullary plasmacytomas [40] or circulating plasma cells [41]. Moreover, methods, such as FISH, may not reveal more complex aberrations (e.g., chromotrypsis or specific gene mutations), which have negative prognostic impact as well.

Daratumumab has limited efficiency in MM patients with plasmacytoma [42–44]. In our analysis, patients with plasmacytoma found at the time of NDMM had somehow better Dara-Rd treatment results than patients, who developed plasmacytoma in disease relapse or progression (RRMM). This interesting finding demonstrates different clinical course of these two entities, even in relapsed setting [45–47]. Our results were, however, influenced by relatively low number of patients with this form of MM.

Clear limitation of our study was a short follow-up of Dara-Rd cohort (median 13.5 months). Based on this limitation, we can more clearly point to patients with limited profit from Dara-Rd treatment than to patients who had the best outcomes. Another important limitation arises from the retrospective and non-randomized character of the analysis and limited cohorts size. For that reason, similarly to other non-randomized RWE studies, especially results in the subgroups should be assessed critically. Other limitation of our study was the absence of valid information of patients’ MRD status while BM evaluations was not routinely done in all patients, as previously described. According to POLLUX results, best treatment results of Dara-Rd regimen were shown in patients achieving CR (42-month PFS rates of 73.6%) [2]. Other study dealing with daratumumab treatment showed achievement of MRD negative status as a most important predictor of treatment success [48].

Taken together, our RWE results emphasize the importance of timing of modern treatment protocols. Dara-Rd treatment in relapsed/refractory setting should be used as soon as possible to maintain best possible effect. Use of this regimen in heavily pretreated or high-risk patients should be individually considered with respect to other treatment options.

## Supplementary Information

Below is the link to the electronic supplementary material.

Supplementary Table 1 Dara-Rd vs. Rd adverse events (DOCX 28 KB)

Supplementary file 2 (PNG 87461 kb)

High resolution image (TIFF 2788 KB)

Supplementary file 3 (PNG 92552 kb)

High resolution image (TIFF 2788 KB)

Supplementary file 4 (PNG 1434 kb)

High resolution image (TIFF 2788 KB)

Supplementary file 5 (PNG 1634 kb)

High resolution image (TIFF 2788 KB)

## Data Availability

Data are available upon request from corresponding author.

## Declarations

### Support

This work was supported by grant of the Ministry of Health of the Czech Republic NU21-03–00076, by grant of Ministry of Health DRO (FNBr, 65269705), by the project National Institute for Cancer Research (Programme EXCELES, ID Project No. LX22NPO5102)—Funded by the European Union – Next Generation EU.
