# Supplementary material for: Daratumumab with lenalidomide and dexamethasone in relapsed or refractory multiple myeloma patients – real world evidence analysis
Source: Ann Hematol. 2023 Apr 24;102(6):1501–11. doi: 10.1007/s00277-023-05188-4 (PMC10182121; doi:10.1007/s00277-023-05188-4)
Supplement: Supplementary file 1 — Supplementary Table 1 Dara-Rd vs. Rd adverse events (DOCX 28 KB) [file 277_2023_5188_MOESM1_ESM.docx]

Supplementary Table 1: Dara-Rd vs. Rd adverse events

| **Neuropathy - grade** | **454** | **100,0%** | **157** | **100,0%** |  |
| --- | --- | --- | --- | --- | --- |
| 0 | 272 | 59,9% | 95 | 60,5% | 0,992 |
| 1 | 117 | 25,8% | 39 | 24,8% |  |
| 2 | 58 | 12,8% | 21 | 13,4% |  |
| 3 | 7 | 1,5% | 2 | 1,3% |  |
| 4 | 0 | 0,0% | 0 | 0,0% |  |
| 5 | 0 | 0,0% | 0 | 0,0% |  |
| **Nausea, vomiting - grade** | **441** | **100,0%** | **156** | **100,0%** |  |
| 0 | 339 | 76,9% | 121 | 77,6% | 0,991 |
| 1 | 63 | 14,3% | 23 | 14,7% |  |
| 2 | 32 | 7,3% | 10 | 6,4% |  |
| 3 | 7 | 1,6% | 2 | 1,3% |  |
| 4 | 0 | 0,0% | 0 | 0,0% |  |
| 5 | 0 | 0,0% | 0 | 0,0% |  |
| **Anorexia - grade** | **438** | **100,0%** | **157** | **100,0%** |  |
| 0 | 321 | 73,3% | 128 | 81,5% | 0,198 |
| 1 | 69 | 15,8% | 21 | 13,4% |  |
| 2 | 39 | 8,9% | 7 | 4,5% |  |
| 3 | 8 | 1,8% | 1 | 0,6% |  |
| 4 | 1 | 0,2% | 0 | 0,0% |  |
| 5 | 0 | 0,0% | 0 | 0,0% |  |
| **Diarrhoea - grade** | **439** | **100,0%** | **171** | **100,0%** |  |
| 0 | 336 | 76,5% | 112 | 65,5% | **0,031** |
| 1 | 41 | 9,3% | 21 | 12,3% |  |
| 2 | 57 | 13,0% | 33 | 19,3% |  |
| 3 | 5 | 1,1% | 5 | 2,9% |  |
| 4 | 0 | 0,0% | 0 | 0,0% |  |
| 5 | 0 | 0,0% | 0 | 0,0% |  |
| **Constipation - grade** | **438** | **100,0%** | **156** | **100,0%** |  |
| 0 | 377 | 86,1% | 134 | 85,9% | 1,000 |
| 1 | 39 | 8,9% | 14 | 9,0% |  |
| 2 | 21 | 4,8% | 8 | 5,1% |  |
| 3 | 1 | 0,2% | 0 | 0,0% |  |
| 4 | 0 | 0,0% | 0 | 0,0% |  |
| 5 | 0 | 0,0% | 0 | 0,0% |  |
| **Fatigue - grade** | **452** | **100,0%** | **169** | **100,0%** |  |
| 0 | 172 | 38,1% | 67 | 39,6% | 0,063 |
| 1 | 127 | 28,1% | 51 | 30,2% |  |
| 2 | 119 | 26,3% | 48 | 28,4% |  |
| 3 | 34 | 7,5% | 3 | 1,8% |  |
| 4 | 0 | 0,0% | 0 | 0,0% |  |
| 5 | 0 | 0,0% | 0 | 0,0% |  |
| **Thrombosis/Thrombus/Embolism - grade** | **440** | **100,0%** | **154** | **100,0%** |  |
| 0 | 398 | 90,5% | 142 | 92,2% | 0,698 |
| 1 | 10 | 2,3% | 2 | 1,3% |  |
| 2 | 16 | 3,6% | 3 | 1,9% |  |
| 3 | 15 | 3,4% | 7 | 4,5% |  |
| 4 | 1 | 0,2% | 0 | 0,0% |  |
| 5 | 0 | 0,0% | 0 | 0,0% |  |
| **Infection - grade** | **458** | **100,0%** | **179** | **100,0%** |  |
| 0 | 203 | 44,3% | 44 | 24,6% | **<0.001** |
| 1 | 40 | 8,7% | 17 | 9,5% |  |
| 2 | 136 | 29,7% | 84 | 46,9% |  |
| 3 | 64 | 14,0% | 26 | 14,5% |  |
| 4 | 11 | 2,4% | 3 | 1,7% |  |
| 5 | 4 | 0,9% | 5 | 2,8% |  |
| **Thrombocytopenia - grade** | **457** | **100,0%** | **153** | **100,0%** |  |
| 0 | 155 | 33,9% | 46 | 30,1% | 0,696 |
| 1 | 161 | 35,2% | 64 | 41,8% |  |
| 2 | 61 | 13,3% | 18 | 11,8% |  |
| 3 | 50 | 10,9% | 15 | 9,8% |  |
| 4 | 30 | 6,6% | 10 | 6,5% |  |
| 5 | 0 | 0,0% | 0 | 0,0% |  |
| **Neutropenia - grade** | **453** | **100,0%** | **157** | **100,0%** |  |
| 0 | 119 | 26,3% | 32 | 20,4% | **<0.001** |
| 1 | 119 | 26,3% | 16 | 10,2% |  |
| 2 | 98 | 21,6% | 29 | 18,5% |  |
| 3 | 88 | 19,4% | 61 | 38,9% |  |
| 4 | 28 | 6,2% | 19 | 12,1% |  |
| 5 | 1 | 0,2% | 0 | 0,0% |  |
| **Anemia - grade** | **460** | **100,0%** | **154** | **100,0%** |  |
| 0 | 65 | 14,1% | 30 | 19,5% | 0,284 |
| 1 | 163 | 35,4% | 55 | 35,7% |  |
| 2 | 131 | 28,5% | 46 | 29,9% |  |
| 3 | 88 | 19,1% | 20 | 13,0% |  |
| 4 | 13 | 2,8% | 3 | 1,9% |  |
| 5 | 0 | 0,0% | 0 | 0,0% |  |
| **Rash (exanthema) - grade** | **352** | **100,0%** | **158** | **100,0%** |  |
| 0 | 319 | 90,6% | 127 | 80,4% | **0,008** |
| 1 | 15 | 4,3% | 15 | 9,5% |  |
| 2 | 15 | 4,3% | 15 | 9,5% |  |
| 3 | 3 | 0,9% | 1 | 0,6% |  |
| 4 | 0 | 0,0% | 0 | 0,0% |  |
| 5 | 0 | 0,0% | 0 | 0,0% |  |
| **IRR (infusion related reaction) - grade** | **210** | **100,0%** | **152** | **100,0%** |  |
| 0 | 210 | 100,0% | 110 | 72,4% | **<0.001** |
| 1 | 0 | 0,0% | 10 | 6,6% |  |
| 2 | 0 | 0,0% | 26 | 17,1% |  |
| 3 | 0 | 0,0% | 6 | 3,9% |  |
| 4 | 0 | 0,0% | 0 | 0,0% |  |
| 5 | 0 | 0,0% | 0 | 0,0% |  |
| **Dyspnoe - grade** | **92** | **100,0%** | **104** | **100,0%** |  |
| 0 | 79 | 85,9% | 79 | 76,0% | 0,161 |
| 1 | 8 | 8,7% | 12 | 11,5% |  |
| 2 | 5 | 5,4% | 13 | 12,5% |  |
| 3 | 0 | 0,0% | 0 | 0,0% |  |
| 4 | 0 | 0,0% | 0 | 0,0% |  |
| 5 | 0 | 0,0% | 0 | 0,0% |  |
| **Decompensated diabetes mellitus - grade** | **92** | **100,0%** | **101** | **100,0%** |  |
| 0 | 86 | 93,5% | 91 | 90,1% | 0,491 |
| 1 | 2 | 2,2% | 7 | 6,9% |  |
| 2 | 3 | 3,3% | 2 | 2,0% |  |
| 3 | 1 | 1,1% | 1 | 1,0% |  |
| 4 | 0 | 0,0% | 0 | 0,0% |  |
| 5 | 0 | 0,0% | 0 | 0,0% |  |
| **Hepatotoxicity - grade** | **90** | **100,0%** | **106** | **100,0%** |  |
| 0 | 82 | 91,1% | 93 | 87,7% | 0,229 |
| 1 | 4 | 4,4% | 10 | 9,4% |  |
| 2 | 2 | 2,2% | 3 | 2,8% |  |
| 3 | 2 | 2,2% | 0 | 0,0% |  |
| 4 | 0 | 0,0% | 0 | 0,0% |  |
| 5 | 0 | 0,0% | 0 | 0,0% |  |
