# Supplementary figures and images for: Daratumumab with lenalidomide and dexamethasone in relapsed or refractory multiple myeloma patients – real world evidence analysis
Source: Ann Hematol. 2023 Apr 24;102(6):1501–11. doi: 10.1007/s00277-023-05188-4 (PMC10182121; doi:10.1007/s00277-023-05188-4)

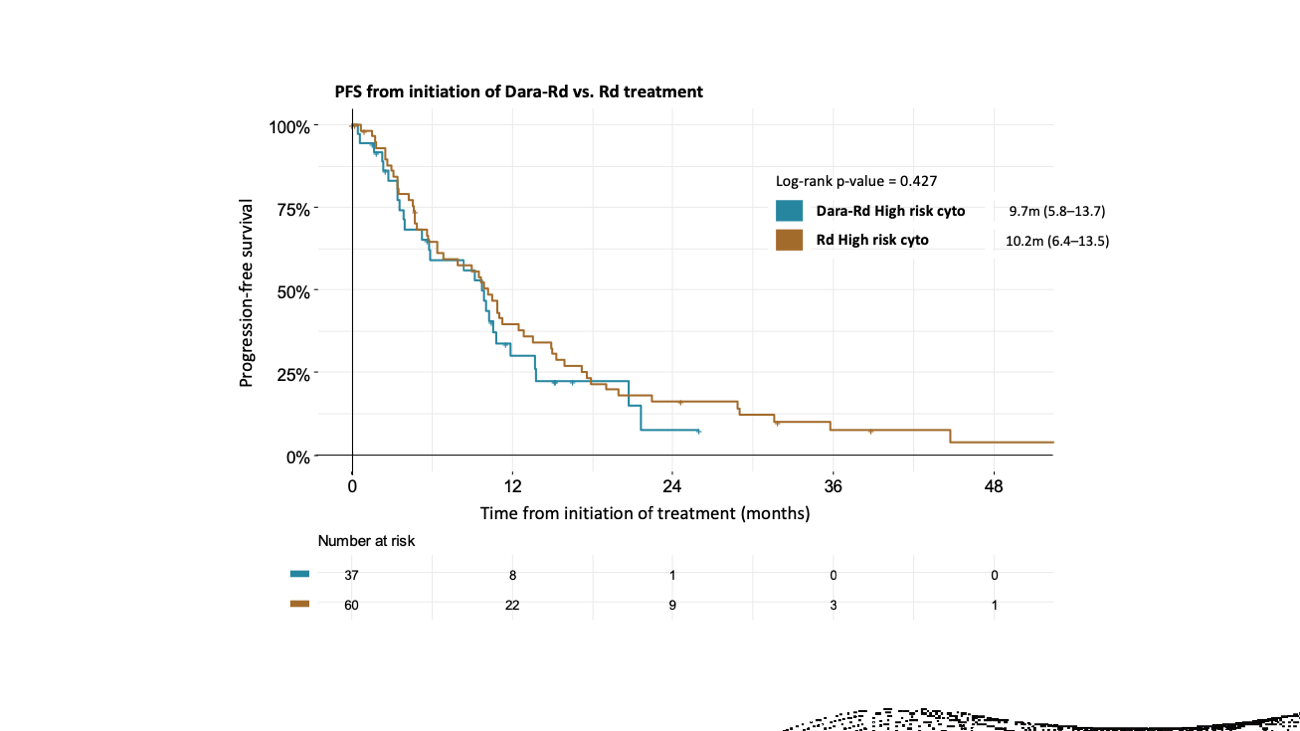

Supplement: Supplementary file 2 — (PNG 87461 kb) [file 277_2023_5188_Fig4_ESM.png]

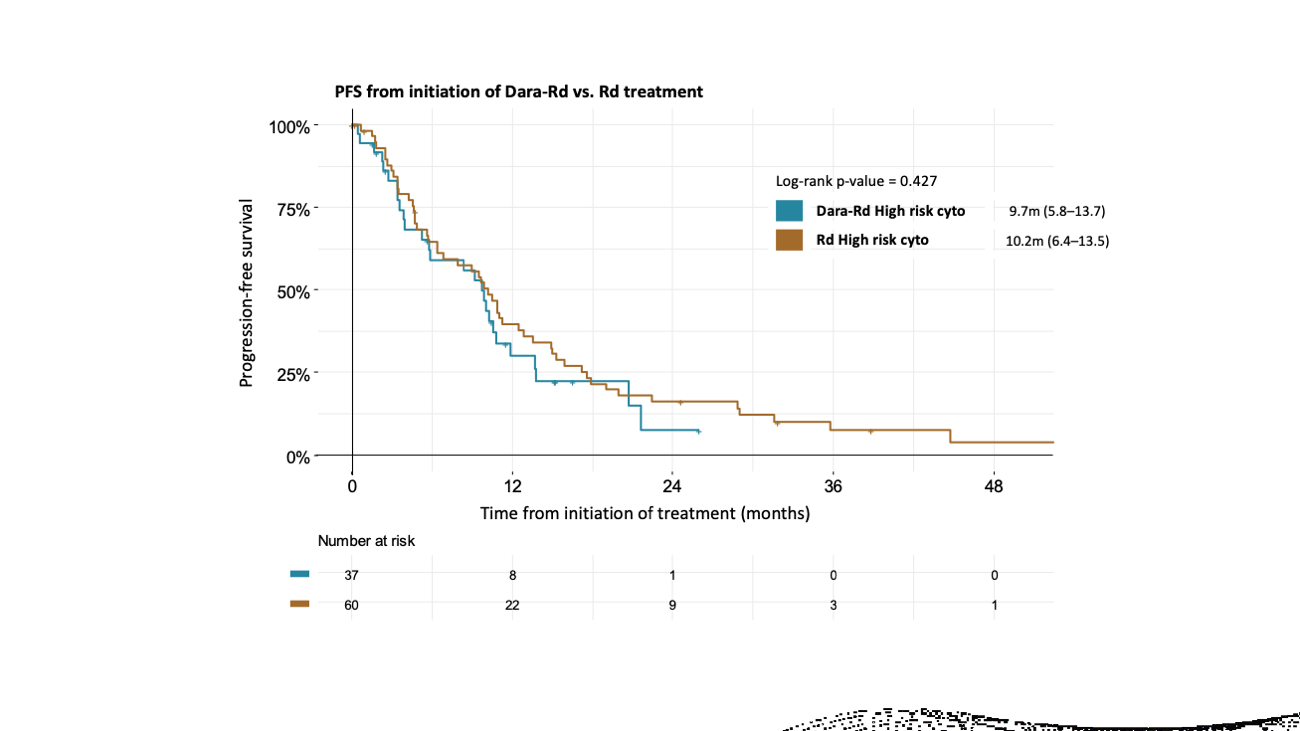

Supplement: Supplementary file 3 — High resolution image (TIFF 2788 KB) [file 277_2023_5188_MOESM2_ESM.tiff]

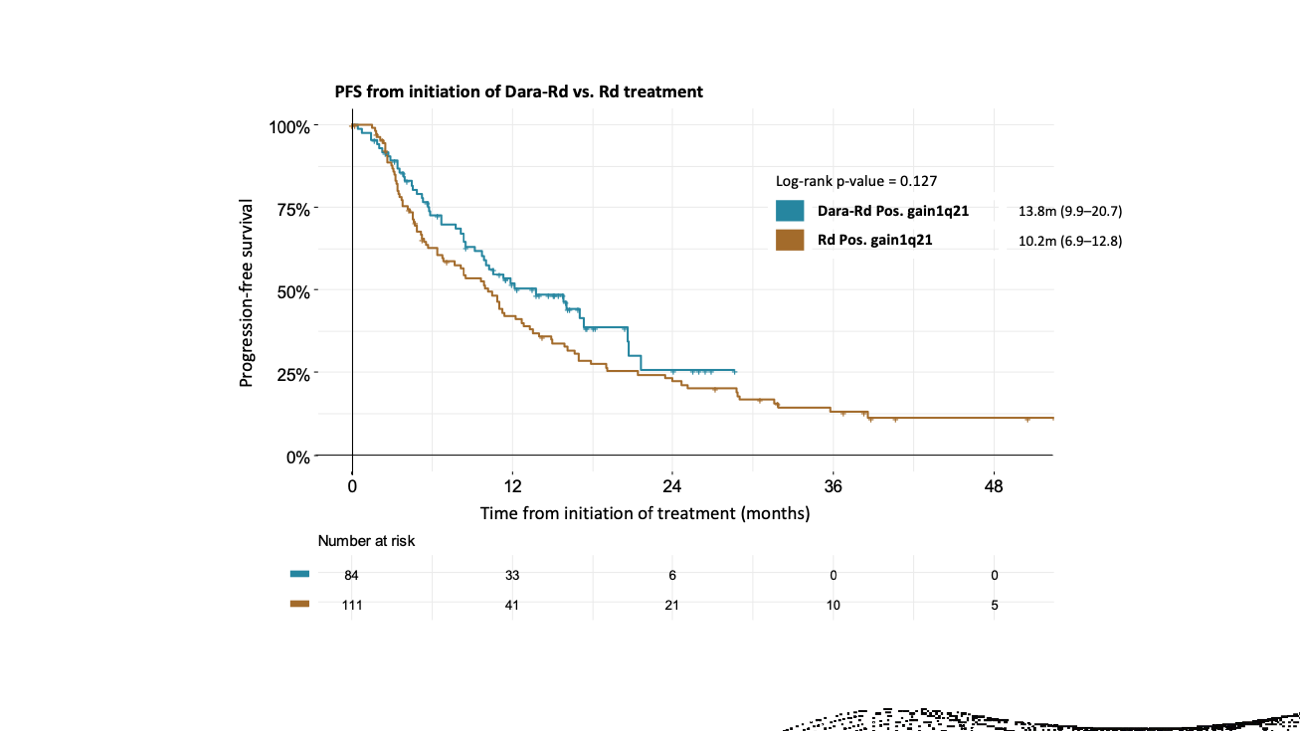

Supplement: Supplementary file 4 — Supplementary file 3 (PNG 92552 kb) [file 277_2023_5188_Fig5_ESM.png]

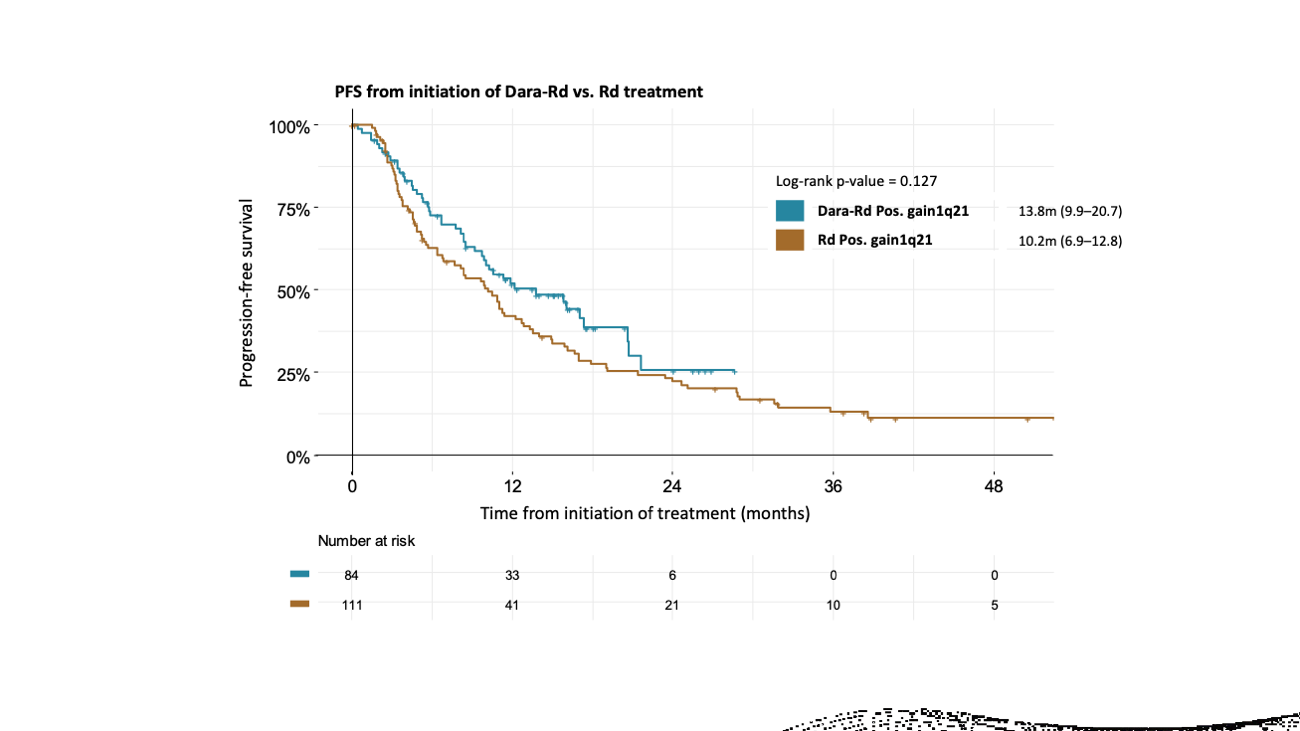

Supplement: Supplementary file 5 — High resolution image (TIFF 2788 KB) [file 277_2023_5188_MOESM3_ESM.tiff]

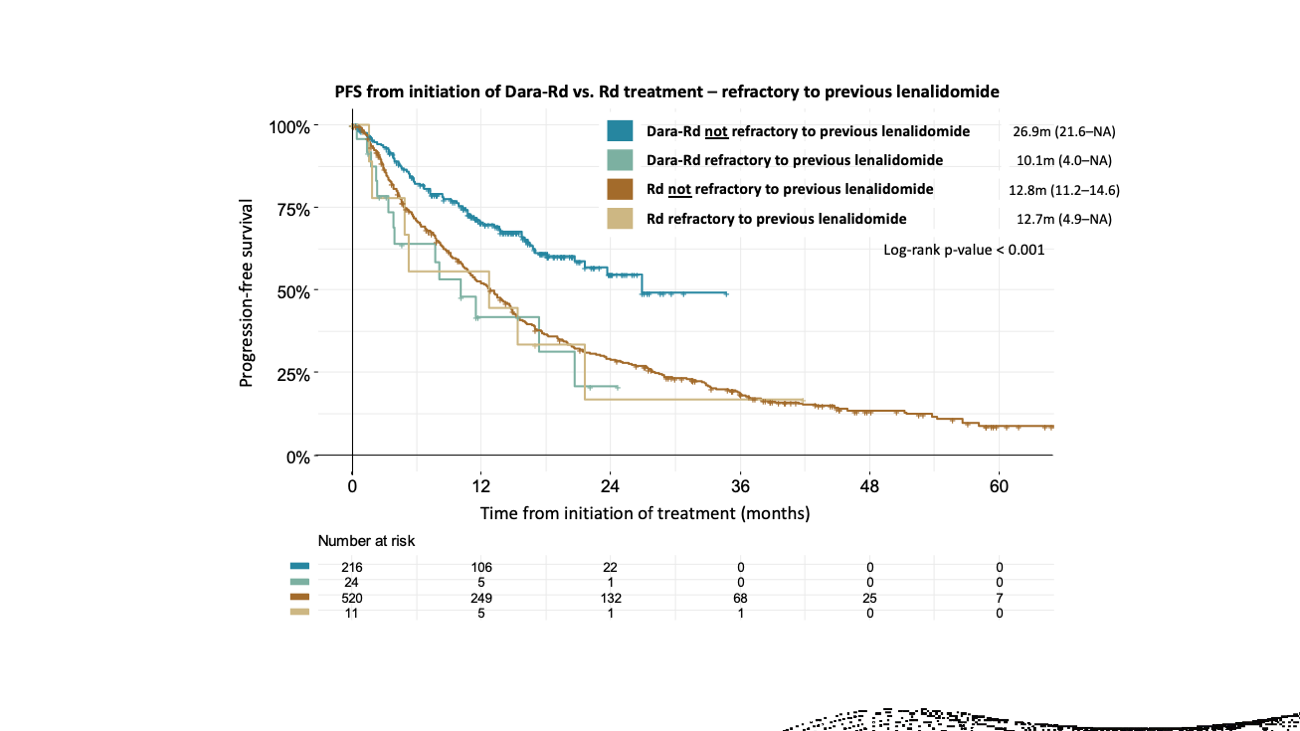

Supplement: Supplementary file 6 — Supplementary file 4 (PNG 1434 kb) [file 277_2023_5188_Fig6_ESM.png]

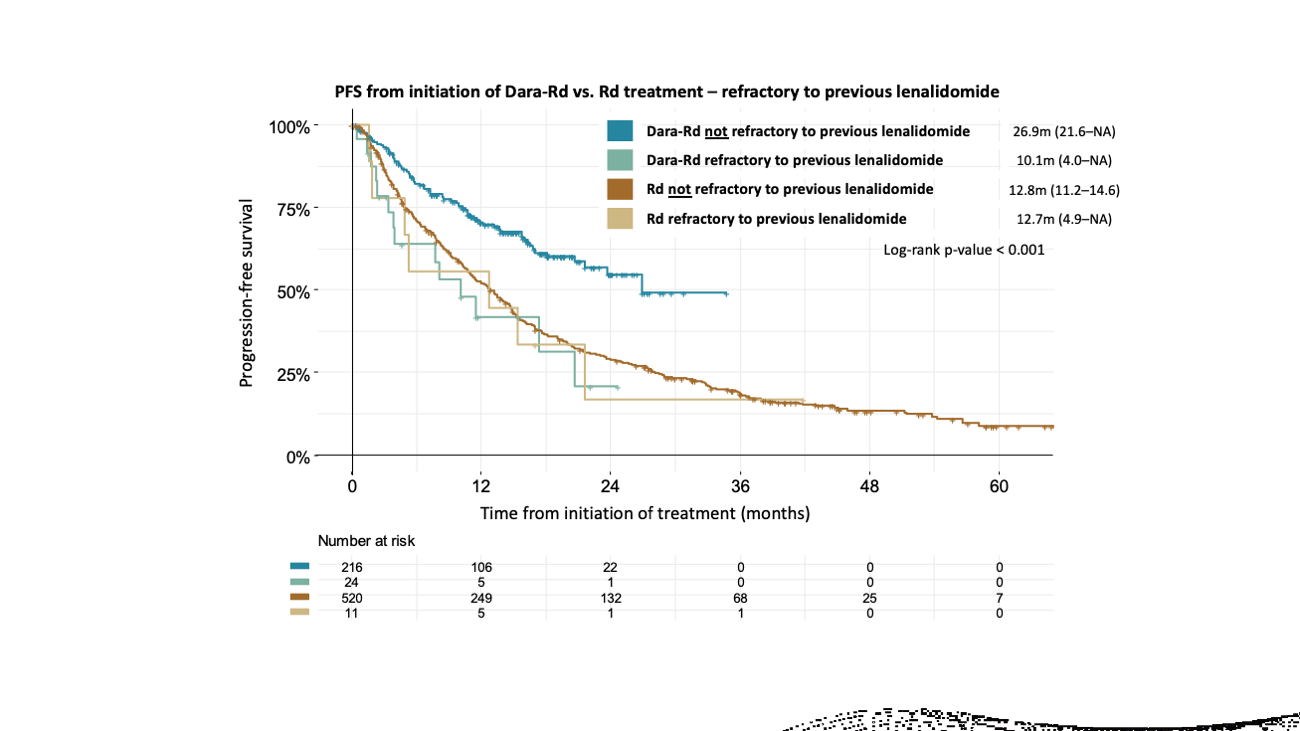

Supplement: Supplementary file 7 — High resolution image (TIFF 2788 KB) [file 277_2023_5188_MOESM4_ESM.tiff]

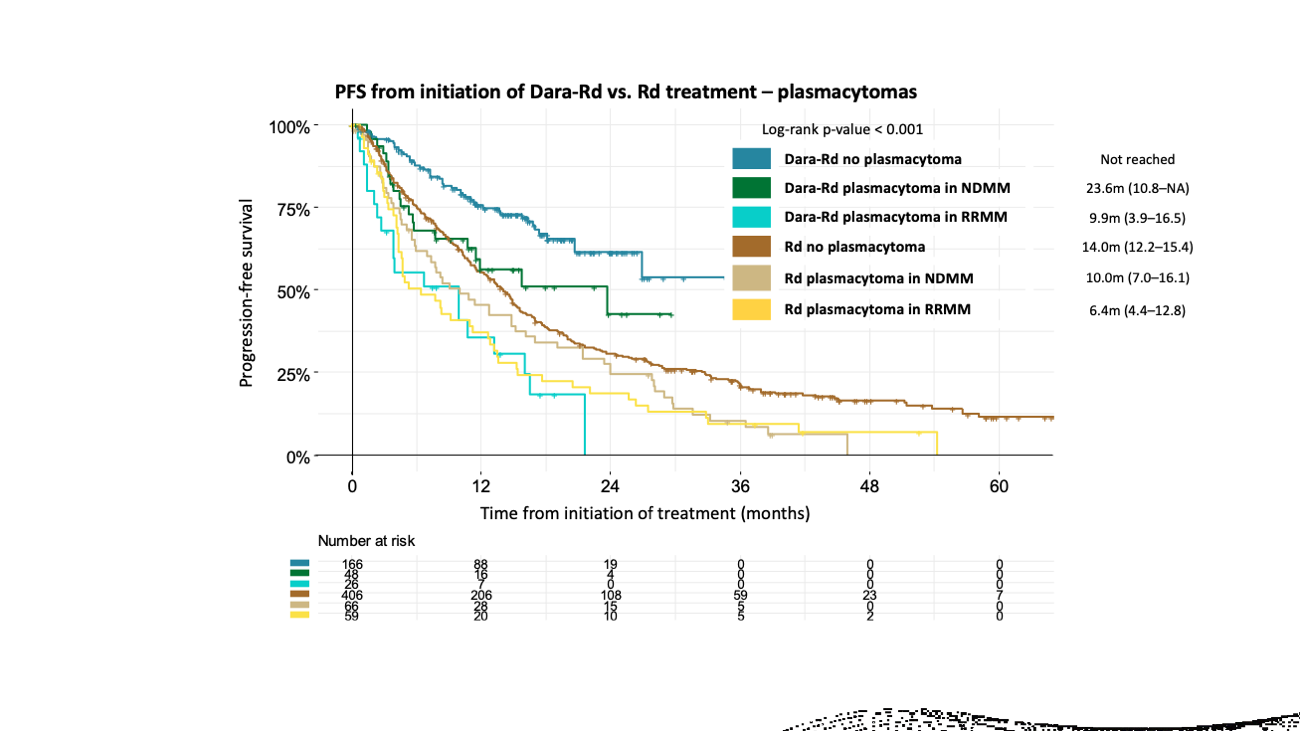

Supplement: Supplementary file 8 — Supplementary file 5 (PNG 1634 kb) [file 277_2023_5188_Fig7_ESM.png]

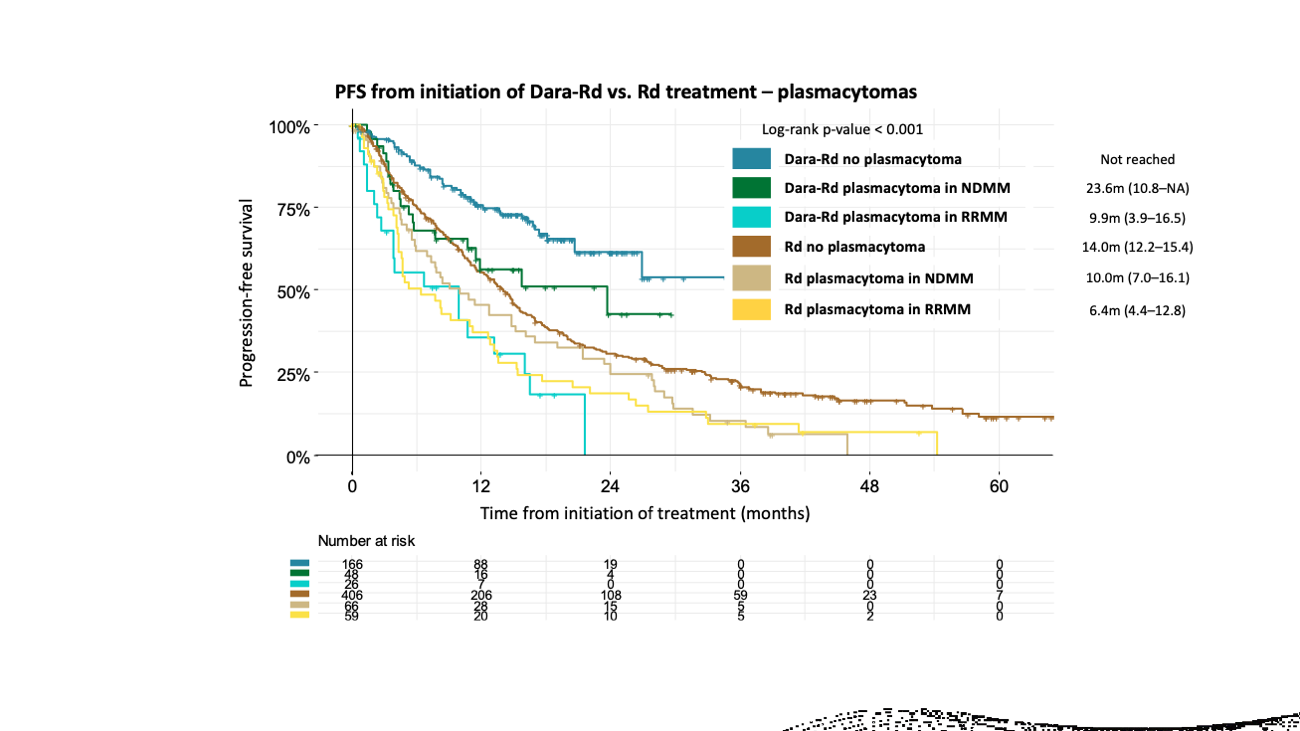

Supplement: Supplementary file 9 — High resolution image (TIFF 2788 KB) [file 277_2023_5188_MOESM5_ESM.tiff]
